# Autophagy: one more Nobel Prize for yeast

**DOI:** 10.15698/mic2016.12.544

**Published:** 2016-12-05

**Authors:** Andreas Zimmermann, Katharina Kainz, Aleksandra Andryushkova, Sebastian Hofer, Frank Madeo, Didac Carmona-Gutierrez

**Affiliations:** 1 Institute of Molecular Biosciences, NAWI Graz, University of Graz, Graz, Austria.; 2 BioTechMed Graz, Graz, Austria.

**Keywords:** yeast, autophagy, ATG, mitophagy

## Abstract

The recent announcement of the 2016 Nobel Prize in Physiology or Medicine, awarded to Yoshinori Ohsumi for the discoveries of mechanisms governing autophagy, underscores the importance of intracellular degradation and recycling. At the same time, it further cements yeast, in which this field decisively developed, as a prolific model organism. Here we provide a quick historical overview that mirrors both the importance of autophagy as a conserved and essential process for cellular life and death as well as the crucial role of yeast in its mechanistic characterization.

More than half a century after the discovery of bulk digestion of cellular components by lysosomes, termed “autophagy” 1,2, the year 2016 marks the latest milestone in the elucidation of this degradation process: in December, Yoshinori Ohsumi will be officially awarded the 2016 Nobel Prize in Physiology or Medicine “for his discoveries of mechanisms for autophagy”. This distinction underscores the broad impact of autophagy research over the last decades. Autophagy, which can target specific proteins, protein aggregates, or even whole organelles (reviewed in 3), has been shown to be involved in various physiological and pathological processes, such as cancer, neurodegeneration, and ultimately the regulation of organismal lifespan (reviewed in 4). While today, autophagic mechanisms are studied in a broad spectrum of organisms, one of them deserves distinguished mention for having been pioneering and still being instrumental in clarifying how autophagy is regulated: the budding yeast, *Saccharomyces cerevisiae*. In this December issue of *Microbial Cell*, we thus pay tribute to the importance of this catabolic process and to yeast as a means to elucidate it by featuring a review article by Daniel Klionsky 5, one of the researchers, who have deeply coined the field. Of note, after 2001, 2006, 2009, and 2013, this year’s Nobel Prize adds to the strikingly high number of awards conceded during the past 15 years to honor work performed in yeast.

In the late 1980s, autophagy research had met a dead end. Hitherto, researchers heavily relied on electron microscopy to identify morphological hallmarks of autophagy, most prominently double-membraned autophagosomes sequestering cellular components and their fusion with lysosomes. However, quantitative assessment of autophagic flux was difficult, because no established marker metabolites or proteins were available. A model organism that allowed screening for autophagic markers as well as the genetic constituents for autophagy, was therefore much needed. In 1992, Y. Ohsumi demonstrated the accumulation of autophagosomes in yeast vacuoles (the analog of mammalian lysosomes) upon mutation of vacuolar proteases 6. The same year, D. Klionsky identified the cytoplasm-to-vacuole (CVT) pathway in yeast 7, corroborating the suitability of this unicellular organism to investigate lysosomal degradation processes.

Remarkably, autophagy in yeast - like in mammalian cells - seemed to respond to starvation conditions, and defects in autophagy could soon be linked to decreased viability under nutrient scarcity. Making use of this effect, 15 autophagy-defective yeast mutants could be isolated from a genetic screen, leading to the discovery of the so-called autophagy-related genes (ATGs) in 1993 8. Only little thereafter, in 1995, Noda *et al.* introduced vacuolar alkaline phosphatase (ALP) activity as the first quantitative, biochemical assay to monitor autophagic activity in yeast 9. A few years later, the ubiquitination-like Atg12p and Atg8p conjugation systems could be identified 10,11,12. Thus, yeast was being instrumental to explore and delineate the mechanistic frame, in which autophagy operates. The discovery of autophagy induction upon inhibition of the nutrient-responsive TOR kinase by rapamycin in 1998 finally embedded autophagy in the physiological response to starvation and paved the way for pharmacological autophagy activation 13. Mechanistically, TOR phosphorylates components of the Atg1p-initiation complex (in particular Atg13p) and thereby inhibits autophagy induction 14. Importantly, mammalian TOR has been implicated in a plethora of diseases (reviewed in 15). The effective haploid yeast system, which allowed various combinations of gene deletions in an unprecedented manner for autophagy research, helped discover further elements of the autophagic machinery, among them the yeast analog of the Beclin-1 complex 16. This phosphatidylinositol 3-kinase (PI-3K) complex is essential for membrane retrieval and autophagosome formation (reviewed in 17). Indeed, PI-3K inhibitors had widely been used as autophagy inhibitors 18,19.

Importantly, the growing field of bioinformatics soon confirmed the evolutionary conservation of ATGs between yeast and humans, e.g. the Atg5-Atg12 and the Atg8-PE conjugation systems 20,21. This circumstance corroborated the status of yeast as a valid model organism for autophagy research. After the autophagic core machinery had been extensively characterized, yeast was used to identify key players of selective autophagic degradation pathways, such as autophagy of mitochondria (termed “mitophagy”), which is mediated by Atg32p 22 and, for instance, has broad implications in the aging process 23,24,25. Other selective autophagy routes, including pexophagy (peroxisomes) or ER-phagy (endoplasmic reticulum) were also first characterized at the molecular level in yeast 26,27. Recently, the selective autophagic degradation of specific proteins, such as the fatty acid synthase has moved into the spotlight 28. In fact, it is tempting to speculate that autophagy not only reshapes cells at the organelle level, but also specifically depletes key regulatory proteins to prime cells for starvation periods. Thereby, pharmacological autophagy inducers such as rapamycin or spermidine, which was also originally identified in yeast 29, might mimic nutrient depletion and thus exert their longevity-promoting effects 30.

**Figure 1:**
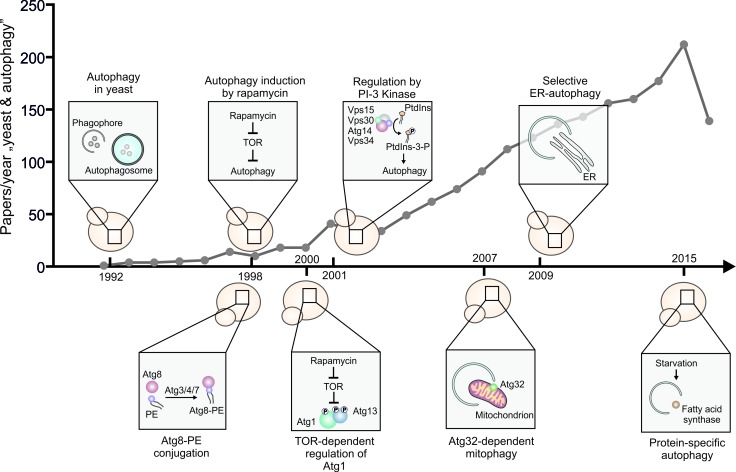
FIGURE 1: A selection of milestones in autophagy research that were first described in yeast and the number of scientific articles per year retrieved in a Pubmed search using the query terms “yeast” and “autophagy”. PE, phosphatidylethanolamine.

Today, the mechanistic framework of autophagy is well characterized, in large parts due to studies performed in yeast. Nevertheless, there are many unanswered questions, including the site of autophagosome formation (reviewed in 31), the role of non-canonical autophagy, which can bypass proteins of the core autophagic machinery 32, the functional connection of the autophagic process with metabolism 33,34, and the exact interplay between autophagy and programmed cell death pathways (reviewed in 35). Extrapolating from the rich history of autophagy research in yeast (Figure 1), we can expect more answers to come from our unicellular buddy.
